# Reducing the Morbidity Associated With Incorrect Pediatric Tracheostomy Tube Placement: A Quality Improvement Initiative

**DOI:** 10.7759/cureus.81619

**Published:** 2025-04-02

**Authors:** Alisha R Pershad, Maria Peña, Habib Zalzal

**Affiliations:** 1 School of Medicine and Health Sciences, George Washington University, Washington, USA; 2 Division of Otolaryngology, Children's National Hospital, Washington, USA

**Keywords:** pediatric airway, pediatric otolaryngology, quality improvement, tracheostomy care, tracheostomy tube

## Abstract

Introduction: Despite an increase in pediatric patients being discharged with a tracheostomy tube (TT), morbidity and mortality rates remain considerable. The aim of this quality improvement (QI) project is to reduce the proportion of tracheostomy documentation errors per 1000 tracheostomy patients by 50% or more, and sustain this for six months.

Methods: Using the Model for Improvement and a Plan-Do-Study-Act (PDSA) cycle, a key driver diagram (KDD) identified challenges in accessing accurate TT information within the electronic medical record (EMR). EMR representatives created an alert for any patient with a TT diagnosis code to provide immediate access to tracheostomy information. Assessment of this intervention was conducted using descriptive statistics and QI control charts.

Results: Prior to intervention, an average of 56 different tracheostomy patients per year were evaluated, and 15 events were recorded. Upon implementation of the alert, there was one safety event in the 180-day pilot period. Since the initial PDSA cycle, there have been two TT documentation events, improving the average from one error in every 40 patients seen over a 28-month period to one error in every 137 patients seen over a 24-month period (and counting).

Conclusion: Increased access to accurate TT information in the EMR demonstrated an increased interval between events. Future work includes routinely tracking events as a new metric to follow and implementing other interventions from the KDD for a multi-interventional approach to the global aim. We recommend that organizations implement this straightforward approach to dramatically reduce untoward outcomes with catastrophic potential.

## Introduction

Advances in medicine have enabled children who normally would not have been able to live without ventilatory support to go home with the assistance of a tracheostomy tube device. While there is an increase in the number of patients stable enough to be discharged with a tracheostomy, late postoperative complications remain considerable, including granuloma formation, accidental decannulation, and airway obstruction [1,2]. Mortality rates are as high as 6% among the pediatric population [3]. Many of these adverse events are preventable or can be mitigated with appropriate and well-informed care [4]. Previous literature suggests that morbidity and mortality rates associated with tracheostomy care can be reduced by education and training of health professionals and caregivers [2,3,5-8]. Specifically, single-institutional reports on their tracheostomy care experiences highlight the importance of comprehensive and holistic training programs to improve outcomes [5,6]. Tracheostomy care training has been shown to be an effective intervention in helping providers understand how to perform tracheostomy tube changes in routine and emergency circumstances and how to select the appropriate tracheostomy tube and equipment needed in these situations [7].

Challenges to pediatric tracheostomy care have been identified in semi-structured interviews with health professionals and families at a single institution in England; top-ranking challenges include inadequate and irregular training, differences in knowledge and familiarity among health providers in different specialties, and limited contact with children with tracheostomies [9]. Tracheostomy changes are often performed urgently or emergently by non-otolaryngology providers. However, when clinicians do not have access to the otolaryngology service’s documentation, an incorrect size or type of tracheostomy can be inserted. Concordance of documentation of the appropriate size and type of tracheostomy tube between the otolaryngologist and other clinicians who are involved in management is important to reduce the potential for errors across the care continuum.

The global aim of this quality improvement (QI) initiative was to reduce late tracheostomy complications. Specifically, the aim of our QI initiative was to decrease the tracheostomy tube documentation error rate in children with tracheostomy tubes from a 2020-2022 baseline incidence of 2.5% by 50%, or more (less than <1.25%), and sustain for six months. A documentation error was defined as a difference between the type and size of tracheostomy from the otolaryngology service’s recommendation. If successful, the QI initiative would then be extended indefinitely and maintained as a permanent resource.

## Materials and methods

This project is undertaken as a QI Initiative at the Children's National Hospital and does not constitute human subjects research. As such, it was not under the oversight of the Institutional Review Board.

Plan-do-study-act cycles

During the initial stages of the project, several discussions during our institution’s monthly quality assessment (QA) meetings by The Children’s National Tracheostomy Care Team raised suspicion that documentation errors were contributing to morbidity associated with tracheostomy care. The Tracheostomy Care Team is composed of two attending pediatric otolaryngologists, two inpatient tracheostomy nurse practitioners, an outpatient otolaryngology nurse manager, an otolaryngology operating room nurse, and intensive care unit nursing leaders and educators across the inpatient floors and units. This multidisciplinary team has been meeting monthly since its initiation on January 27, 2022. The inception of this QI initiative was based on interest in tracheostomy care by stakeholders involved in this multidisciplinary team in the hospital.

**Figure 1:**
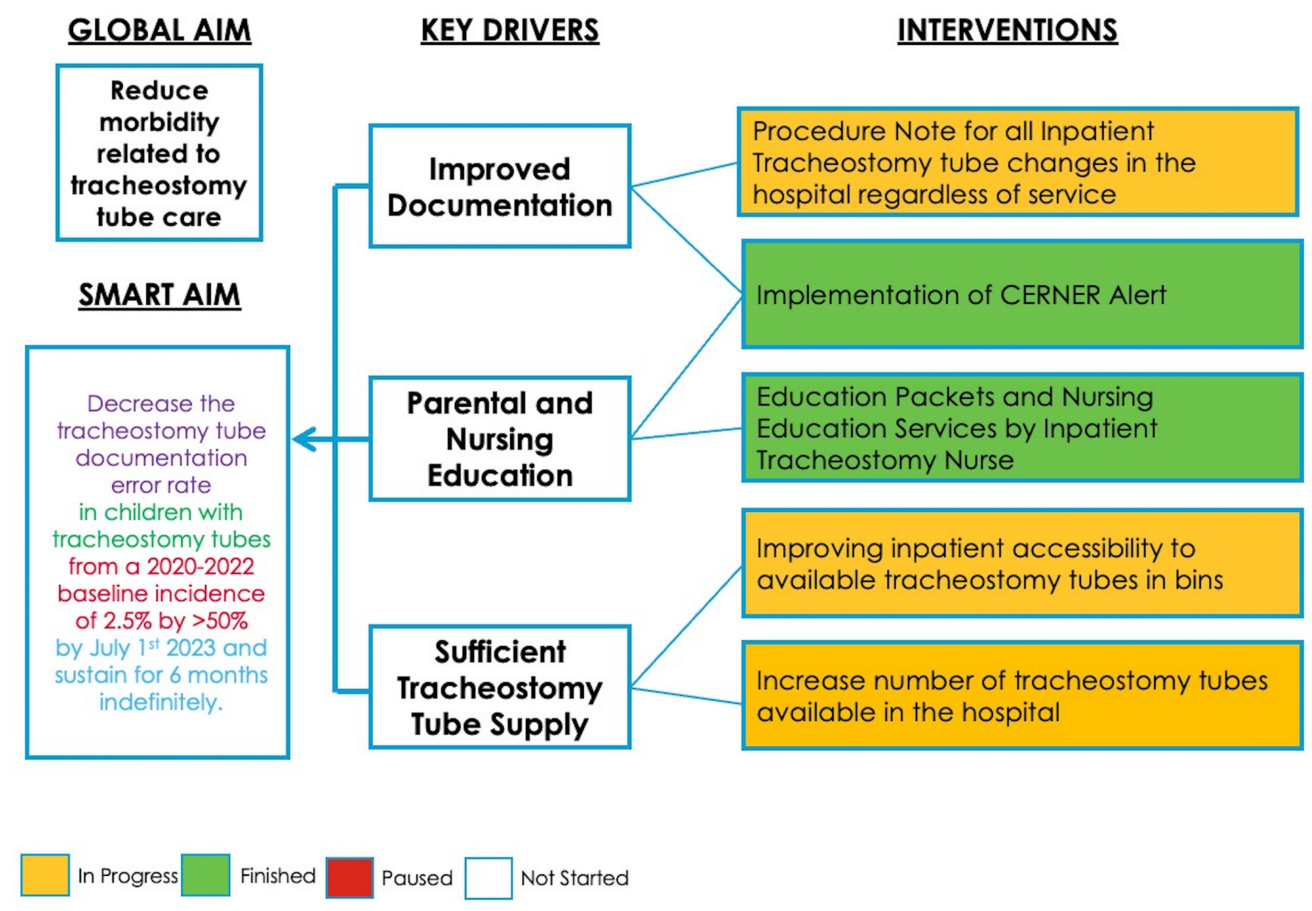
Key driver diagram identifying the following primary drivers of improved documentation, parental and nursing education, and sufficient tracheostomy tube supply to accomplish the aim

A fishbone and key driver diagram analysis was conducted by the Tracheostomy Care Team to determine the possible etiologies of morbidity related to tracheostomy care over the course of the COVID-19 pandemic (August 2020 through December 2021) (Figure 1). One such driver formally identified was documentation errors, which were reported when an indwelling tracheostomy was inconsistent with the Otolaryngology service’s recommendations. The sentiment ascertained from these analyses was that poor access to accurate tracheostomy tube information in the electronic medical record (EMR) led to more errors and increased safety events. At the time of data accumulation, accurate documentation of tracheostomy tubes was only consistently identified in the inpatient nursing notes, operative reports, or otolaryngology clinic notes, which were difficult and time-consuming to find in patients with medically complex histories requiring significant and repeated input from the entire patient care team.

In early 2022, the team reviewed both inpatient and outpatient charts looking for documentation of the types of tracheostomy tubes in place, presence/absence of cuffs, and, if cuffed tubes were present, what volume of air/water was used. The team realized that many tracheostomy tube management decisions were changed outside of the recommendations made by the Otolaryngology service, and moreover, these changes were not being documented. Changes made outside the recommendations made by the Otolaryngology service, due to missing, incomplete, incorrect, or hidden information regarding tracheostomy care, constituted a documentation error.

The attending pediatric otolaryngologists (MP and HZ) proposed a project using the Model for Improvement and a Plan-Do-Study-Act (PDSA) cycle to reduce tracheostomy-related morbidity by reducing documentation errors for tracheostomy patients at our institution [10]. The first step in this methodology, the "plan" stage, involved establishing a specific, measurable goal and developing a proposal to test the initiative.

**Figure 2:**
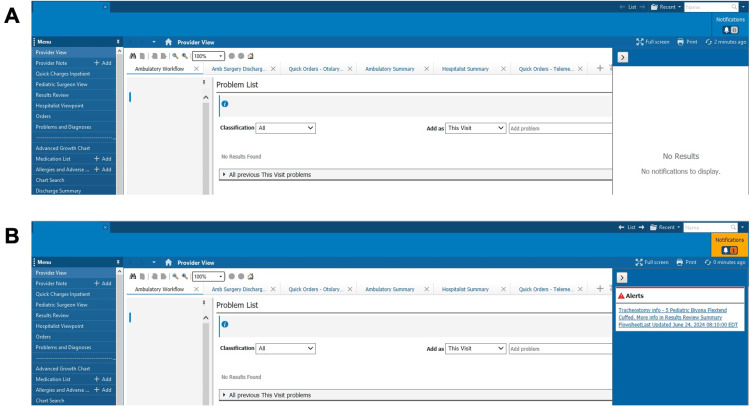
(A) Example patient medical record within Cerner without tracheostomy alert. (B) Example patient medical record within Cerner with tracheostomy alert

In February 2022, our team collaborated with our EMR vendor, Cerner Powerchart® (Oracle Health), to develop an alert system for patients with a tracheostomy tube diagnosis code. The alert was designed to appear at the top of the patient's chart upon entry into the medical record. When otolaryngology and nursing staff documented a tracheostomy tube change, Cerner converted this data into an alert (Figure 2). Patients requiring a tracheostomy alert were identified by a physician approving the ICD-10 (10th revision of the International Classification of Diseases) diagnosis code for the presence of a tracheostomy tube (ICD-10 code Z93.0). The alert auto-populated with tracheostomy tube information based on data input by nursing staff under the "Vital Signs" section during medical office visits. Otolaryngology staff were responsible for ensuring the alert's presence, while nursing staff documented the tracheostomy information that populated the alert. The goal was to provide physicians and nurses with immediate access to accurate tracheostomy tube information in the EMR.

This intervention was implemented in December 2022. To address documentation errors, we aimed to reduce the proportion of errors from our baseline pandemic error rate. The error rate was defined as instances of poor documentation relative to the number of tracheostomy patients seen by the Otolaryngology service. Although not all tracheostomy patients are seen by the Otolaryngology service, this measurement served as a process measure to demonstrate improvement over time. Following the planning phase, the team transitioned to the "study" and "act" stages of the PDSA cycle, assessing interventions to formalize recommendations for EMR alerts.

Statistical analysis

Data was collected and managed using our institutional electronic cloud tools. Statistical evaluation was performed using Microsoft Excel 365® (Microsoft Corporation 2023, Redmond, WA) and QI Macros (Version 2023.04, KnowWare International Inc., Denver, CO). Data was summarized using standard descriptive statistics. Additional analysis using a P-Control Chart was used for monitoring the proportion of events over the pre- and post-intervention periods. A P-chart was chosen as an appropriate choice for documentation to report the proportion of events out of the number of tracheostomy patients seen by the Otolaryngology service due to the low volume and infrequency of tracheostomy documentation errors.

## Results

**Figure 3:**
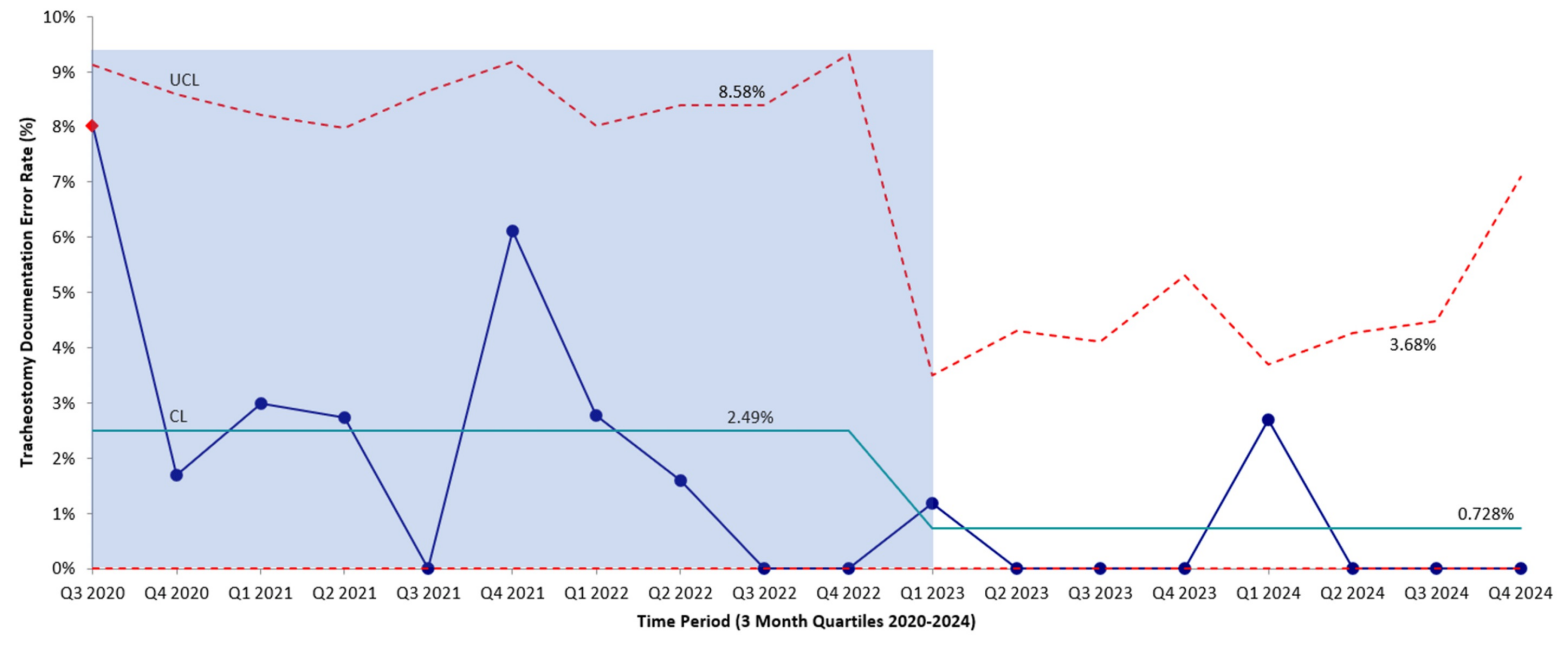
‘P’ Control Chart depicting the tracheostomy documentation error rate per quarter in the pre-intervention and post-intervention period UCL: upper control limit; CL: center line reporting tracheostomy documentation error rate prior to and after implementation of the initiative; QA: quality assessment; Q3 2020 to Q4 2022: pre-intervention phase; Q1 2023 onwards: post-intervention phase.

Before intervention, the number of consults for tracheostomy and tracheostomy-related safety events over 28 months was 601, compared to the 412 patient encounters documented in the post-intervention period. The majority of these consultations were negative, but a total of 108 had an actual safety event (Table 1). In that timeframe, an average of 45 new tracheostomy procedures per year were performed, and 56 different patients with a tracheostomy tube were evaluated by the Otolaryngology service. Looking specifically at tracheostomy tube documentation errors, 15 events were recorded between August 2020 and May 2022 (Figure 3). The average number of days between events was 43.4 days. The longest time interval between safety events was 225 days between April and December 2022, following the implementation of the monthly tracheostomy QA education meetings. Pre-intervention, there were 25 errors per 1000 tracheostomy patients. Post-intervention, the error rate decreased to seven errors per 1000 tracheostomy patients.

**Table 1:** Number of tracheostomy-related safety events and consults received by the otolaryngology department from 2020 through 2022

Safety Event	# of Events
Mortalities	7
Related to tracheostomy tube decannulation	4
Unrelated to tracheostomy tube	3
Bleeding from tracheostoma	27
Unplanned decannulation events	18
Air leak or plugging events	18
Stomal breakdown/false passage formation	16
Tracheostomy tube documentation errors	15

Upon initiation of the medical alert regarding tracheostomy patients, there was only one safety event in the proceeding 180-day period. This safety event occurred during the care of a patient who had not been seen in the hospital in almost 15 months, a consequence of the COVID-19 pandemic and associated barriers to accessing care. Poor access to care led to difficulty in determining which tracheostomy tube was actively being used. Since the initiation of the first PDSA cycle to identify the problems associated with medical documentation errors, there have only been two more tracheostomy tube documentation events (Table 2). The study has continued beyond the 180-day trial period and reported to the Tracheostomy Care Team.

**Table 2:** Data points regarding tracheostomy documentation error events: August 2020-current (December 31, 2024)

Phase of Initiative	# of Events	# of Events per 1000 Patients	Time Period (Months)
Pre-intervention (August 2020-December 31st, 2022)	15	25	28
Post-intervention (implemented January 1st, 2023)	3	7	24

## Discussion

Pediatric intensivists, otolaryngologists, and respiratory technicians, among other specialists, continue to work to improve the care of children with a tracheostomy. This QI initiative is an effort to build on prior work. Compared to prior literature that focuses on implementing educational programs and training days on tracheostomy care to address knowledge gaps [11,12], we target an alternative etiology of tracheostomy care morbidity and observe a sustained improvement.

The principal intervention of creating an EMR alert drove improvement by providing real-time access to tracheostomy care knowledge. Previously at our institution, there was no central area that providers could access to inform the care team that a given patient had a tracheostomy tube and thus be aware of tracheostomy-care-related issues during the patient’s stay. By providing an alert for the providers upon entry to the EMR, the frequency of tracheostomy-related safety events has been reduced, demonstrating initial success in accomplishing the global aim of reducing the morbidity related to tracheostomy tube care.

One key takeaway that facilitated the implementation of our QI initiative was the role of multidisciplinary coordination. Our institution had a previously established multidisciplinary team that met regularly to review and discuss tracheostomy care. The monthly QA meetings focused attention on the morbidity associated with tracheostomy care, which primed providers to identify opportunities for improvement. Previous literature has shown that multidisciplinary teamwork is an important aspect in improving the care of pediatric tracheostomy patients [13-16]. Multidisciplinary teams help with employing standardized protocols; therefore, having this space to discuss documentation errors and identify this variation facilitated the implementation of our QI initiative.

Here we report the results of one intervention, the creation of a smart zone alert in Cerner, as our first PDSA cycle. By the Model for Improvement, PDSA cycles are supposed to repeat and be connected in a cycle of learning and adaptability. We acknowledge that more PDSA cycles are to be tested to apply the intervention across different settings. However, we also endorse that the intervention itself is a straightforward approach to improve communication and patient care, compared to the other interventions identified from the key driver diagram that were more involved. Tracheostomy care has become increasingly complex with the wide range of tubes, associated devices, and customization practices [17]. Pediatric tracheostomies are associated with higher rates of mortality when compared to adult patients, in part due to increased comorbid conditions that require complex care and the need for rapid intervention [18]. Families and healthcare professionals have cited disorganized tracheostomy care and poor communication as obstacles to optimizing outcomes [14]. Having essential information available to a multidisciplinary team of tracheostomy responders, regardless of their status as an airway provider, can mitigate these differences in knowledge and comfort with tracheostomy care.

Future work for testing more PDSA cycles and other interventions at our tertiary academic medical center will be driven by the key driver diagram. Outside of improving documentation, promoting parental and nursing education, and ensuring sufficient tracheostomy tube supply were identified as primary drivers for achieving the global aim. A multi-interventional approach of maintaining the EMR alert while concurrently providing parental and nursing education to build confidence and reduce discrepancies in care among caregivers of pediatric patients with tracheostomy tubes is our intended target.

In addition to expanding the implementation of the previously identified interventions to reduce morbidity associated with tracheostomy care, monitoring of any new developments with our existing Cerner alert protocol will be continued. As this specific initiative is in sustain mode, we noted after the 180-day trial period that there is an error in the EMR process that codes the presence of a gastrostomy tube as triggering the tracheostomy alert in patients who did not even have a tracheostomy. However, due to elevated awareness with the new tracheostomy alert, documentation was not affected for these patients within the patient notes, nor were any clinical errors committed. Furthermore, this error was rapidly identified and corrected within 45 days. 

This work aligns with current professional society aims. In 2013, the American Academy of Otolaryngology-Head and Neck Surgery called for a reduction in the variation of adult and pediatric tracheostomy care among clinicians and institutions [19]. Our initiative addresses this consensus statement by providing immediate access to tracheostomy care information for patients with a tracheostomy tube diagnostic code at the time a provider may need that information. Such an approach is more patient-specific compared to general education modules used to standardize pediatric tracheostomy care.

Key strengths of our particular study are the intervention’s ease of implementation, the study’s clear measures of improvement in the form of documentation errors, and the team’s engagement in multidisciplinary collaboration. Limitations of our study include possible confounding effects. For example, our institution is one that is highly engaged with QI initiatives and highly amenable to change. Simply an increase in attention to the problem, possibly secondary to the Hawthorne effect and concurrent education efforts, may have stimulated some improvement and contributed to the success observed. Prolonged assessment of the success of this intervention, not only on the impact on documentation errors, but also on the impact of patient safety measures, will continue to be a focus at our institution. This study’s generalizability is limited because this methodology has been tested at one site; therefore, we can only comment on the impact of this intervention in this particular resource level with this particular EMR. Scaling this intervention across different EMR systems will inevitably look different and could contribute to alert fatigue among users. Future investigations should include testing this easily implementable initiative across multiple institutions, as scaling up initiatives does not necessarily guarantee success; however, the intervention is straightforward and low-risk.

## Conclusions

An easy-to-implement EMR alert system has been shown to reduce tracheostomy tube documentation errors. Future work for this initiative includes continuing monitoring beyond the 180-day trial period and implementation of other interventions as identified from the key driver diagram for a multi-interventional approach to the global aim. While this intervention does not limit all tracheostomy errors, the intervention can help prevent controllable errors, such as documentation discrepancies. We recommend that all organizations implement a seemingly straightforward approach to dramatically improve untoward outcomes that have catastrophic potential.
